# Supplementary material for: Differential Regulation of MMPs, Apoptosis and Cell Proliferation by the Cannabinoid Receptors CB1 and CB2 in Vascular Smooth Muscle Cells and Cardiac Myocytes
Source: Biomedicines. 2022 Dec 16;10(12):3271. doi: 10.3390/biomedicines10123271 (PMC9775096; doi:10.3390/biomedicines10123271)
Supplement: Supplementary file 1 [file biomedicines-10-03271-s001.zip › biomedicines-2079619-supplementary.pdf]

Supplementary Material

Figure S1

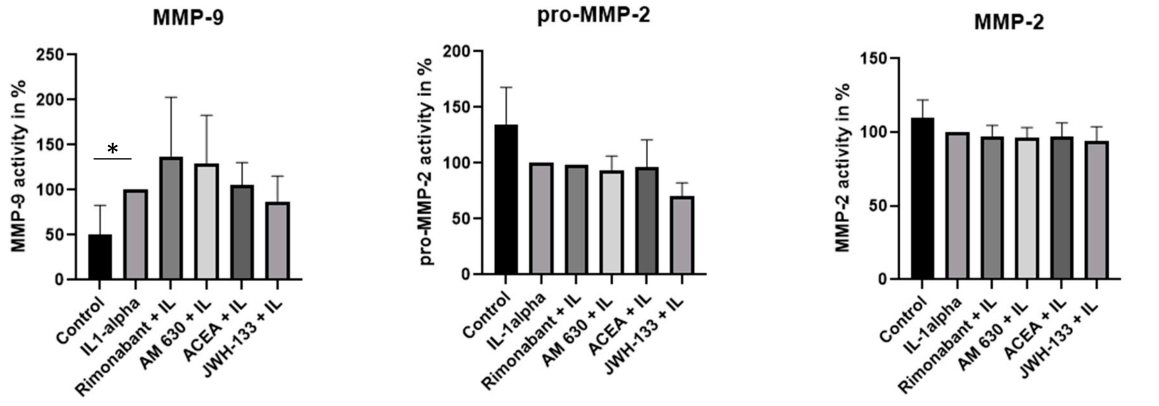

(a) (b) (c)

Figure S1. Effect of Rimonabant, AM630, ACEA and JWH-133 on IL-1α induced secretion of MMP-9 (a), proMMP-2 (b) and MMP-2 (c) in H9c2 cells, 48h after treatment. The graphs represent the densitometric analysis (mean ± SD; n =5–9 for MMP-9 and MMP-2, pro-MMP-2 (Rimonabant n =1; Control, IL1-alpha n =2; ACEA, JWH-133, AM 630 n =3). Statistical analysis performed with t-test with Welchs correction, *$p < 0.05$.

Figure S2

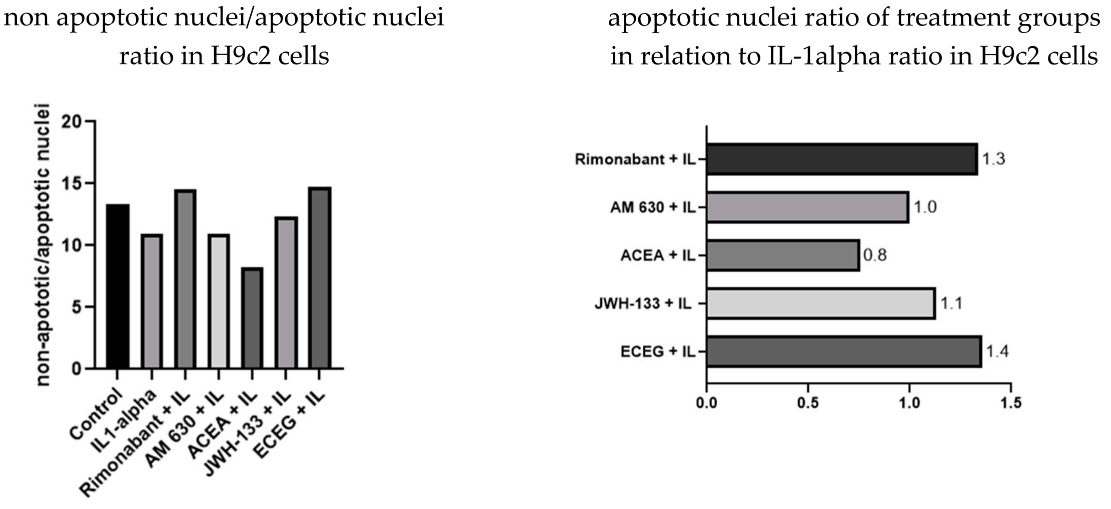

(a) (b)

Figure S2. (a) The ratio of non-apoptotic normal cell nuclei to apoptotic cell nuclei in H9c2 cells 48 h after stimulation with IL-1α (b) The non-apoptotic/apoptotic cell nuclei ratio in relation to IL-1α stimulation. Treatment groups with a ratio over 1.0 have more normal cell nuclei than those in the IL-1α group.

Figure S3

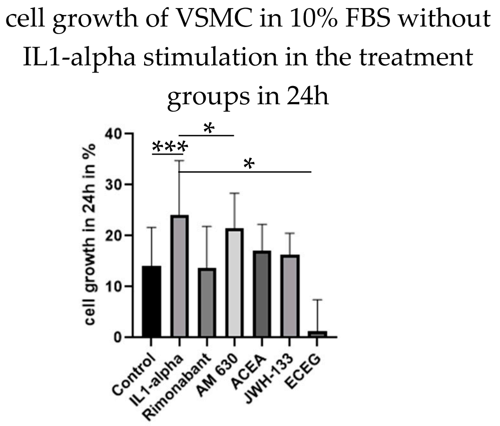

(a)

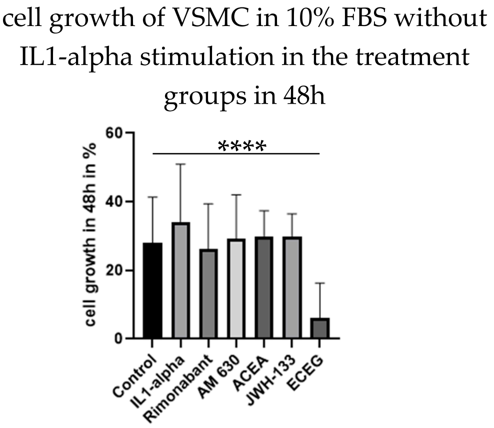

(b)

Figure S3. The growth rate (confluence difference) of VSMCs estimated by IncuCyte live-cell analysis after treatment with compounds without IL-1α stimulation in the treatment groups in 24 h (a) and 48 h (b). Statistical testing was performed using unpaired t-tests between treatment and control group. Significance was expressed when $p < 0.05$; n =5–32 (*$p < 0.05$; ***$p < 0,01$; ****$p < 0.001$).

Figure S4

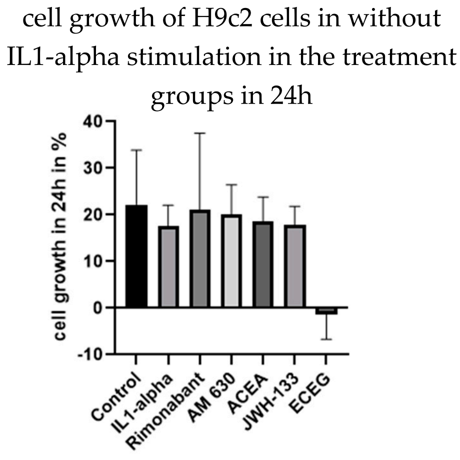

(a)

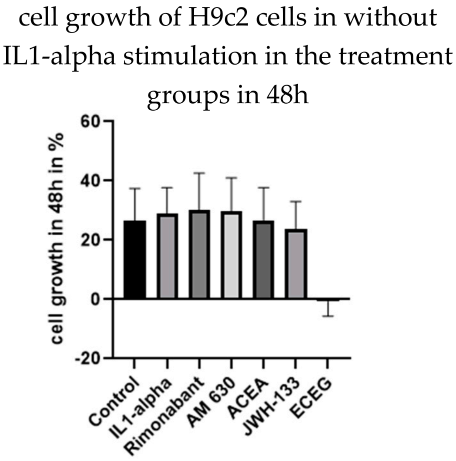

(b)

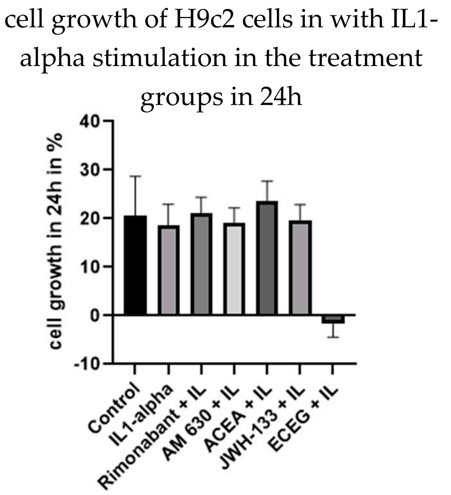

(c)

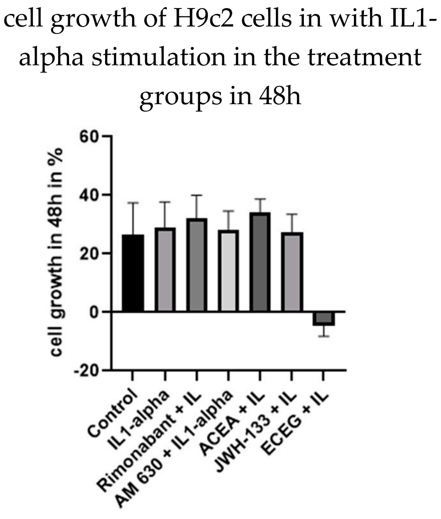

(d)

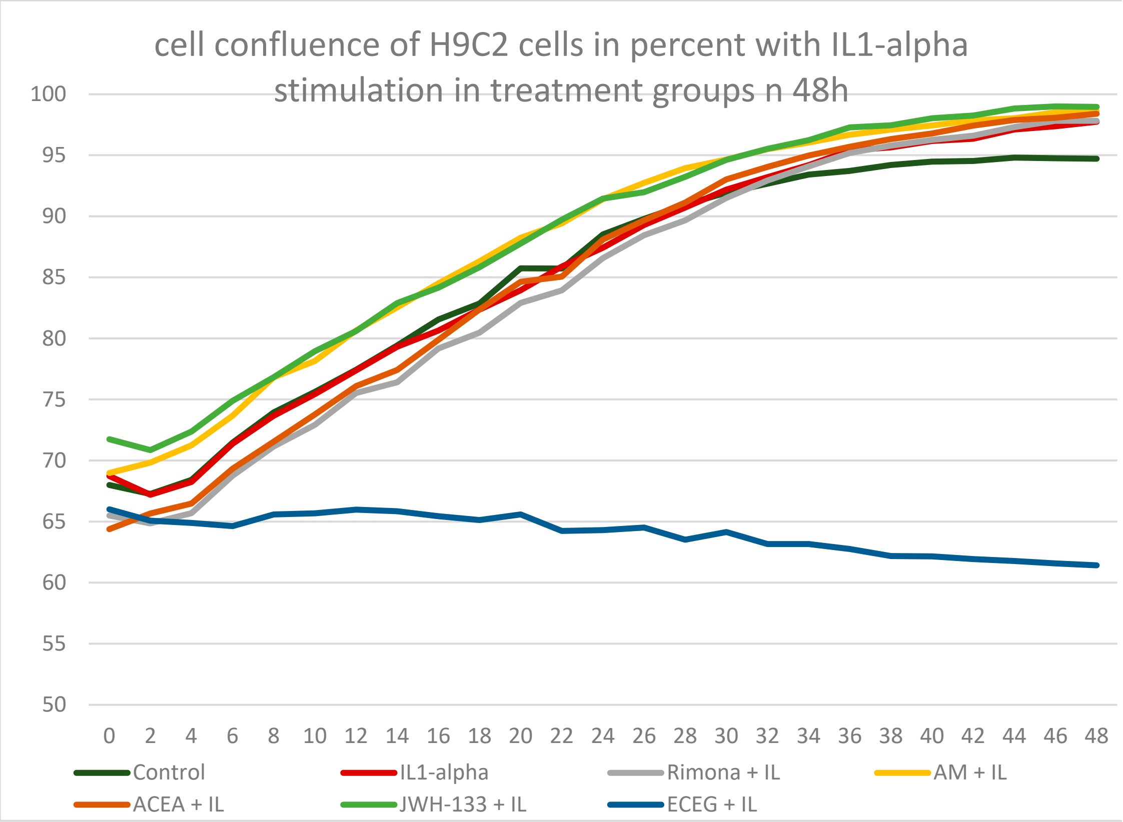

(e)

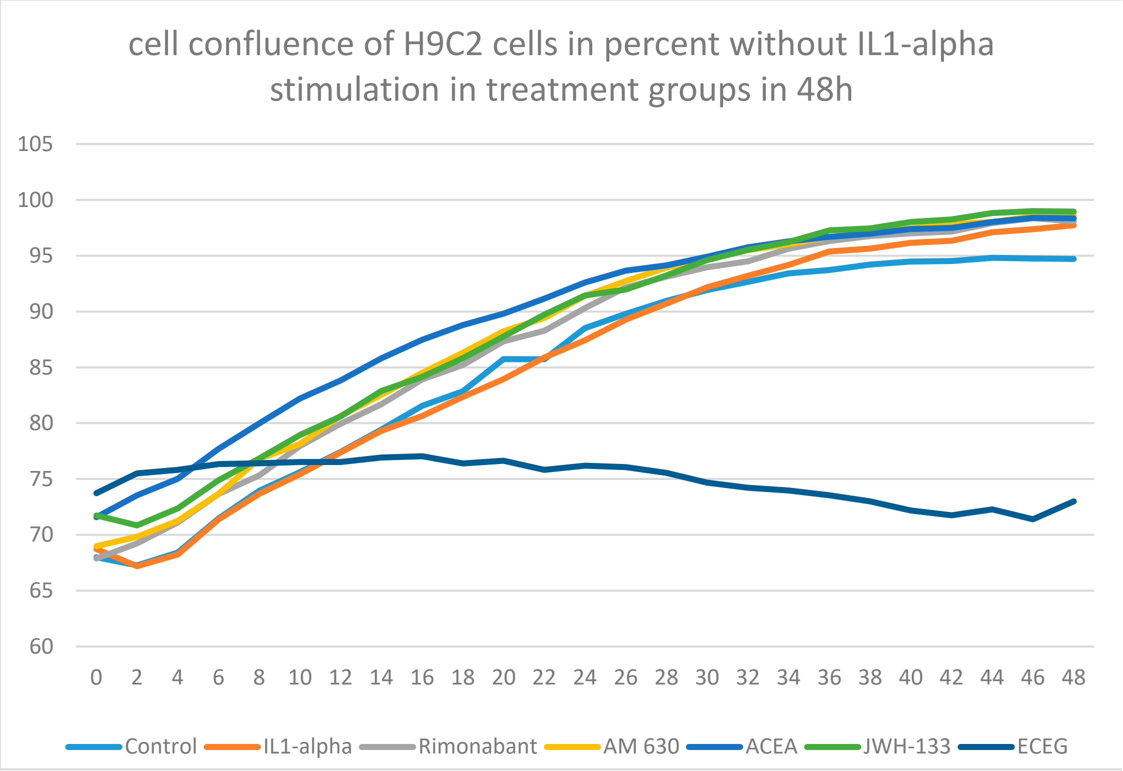

(f)

Figure S4. The growth rate (confluence difference) of H9c2 cells estimated by IncuCyte live-cell analysis after treatment with compounds without IL-1α stimulation in 24 h (a) and 48 h (b) and with IL1-alpha stimulation in 24 h (c) and 48 h (d). (e) and (f) H9c2, cell growth in dynamic. Representative graph obtained from IncuCyte Live Cell Analysis System. Cell proliferation was monitored by analysing the occupied area (% confluence) of cell images over 48 h. Analysis of the IncuCyte images was performed with Incucyte® Analysis Software. The experiment was performed using 10% FBS medium with (f) and without (e) IL-1α stimulation in all treatment groups.

Figure S5

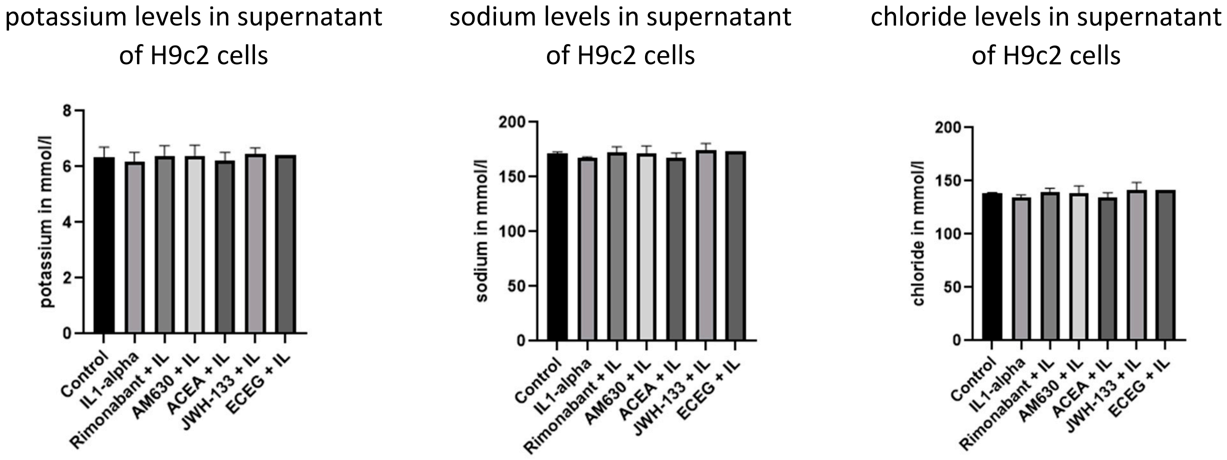

(a) (b) (c)

Figure S5. The concentrations of potassium (a), sodium (b) and chloride (c), measured in cell supernatant of H9c2 cells 48 h after treatments with compounds and IL-1α stimulation.
